# Testing the equivalence of survival distributions using PP- and PPP-plots

**DOI:** 10.1186/1745-6215-14-S1-P106

**Published:** 2013-11-29

**Authors:** Trevor Cox

**Affiliations:** 1 Liverpool University, Liverpool, UK

## 

PP-plots for survival distributions are considered where one survival distribution is plotted against the other. This is seen as another way of visualizing the nature of the relationship between the two survival distributions along with typical Kaplan-Meier plots. For three survival distributions, the PPP-plot is introduced where the survival distributions are plotted against each other in three-dimensions. Two test statistics are defined, based on areas and lengths associated with the PP- and PPP-plots to test the null hypothesis of equivalent survival curves. A simulation exercise has shown that the new tests are worthy competitors to the logrank and Wilcoxon tests. Also illustrated is how the PP-plot can be used to estimate the hazard ratio and also to assess the ratio of hazard functions if proportional hazards are not appropriate. The methods introduced are illustrated on two cancer data sets.

